# Overexpression of caspase 7 is ERα dependent to affect proliferation and cell growth in breast cancer cells by targeting p21^Cip^

**DOI:** 10.1038/oncsis.2016.12

**Published:** 2016-04-18

**Authors:** S Chaudhary, B Madhukrishna, A K Adhya, S Keshari, S K Mishra

**Affiliations:** 1 Cancer Biology Laboratory, Gene Function and Regulation group, Institute of Life Sciences, Nalco Square, Chandrasekharpur, Bhubaneswar, Odisha, India; 2 Department of Pathology, Kalinga Institute of Medical Sciences, KIIT Rd, Chandaka Industrial Estate, Patia, Bhubaneshwar, Odisha, India

## Abstract

Caspase 7 (*CASP7*) expression has important function during cell cycle progression and cell growth in certain cancer cells and is also involved in the development and differentiation of dental tissues. However, the function of CASP7 in breast cancer cells is unclear. The aim of this study was to analyze the expression of CASP7 in breast carcinoma patients and determine the role of *CASP7* in regulating tumorigenicity in breast cancer cells. In this study, we show that the CASP7 expression is high in breast carcinoma tissues compared with normal counterpart. The ectopic expression of *CASP7* is significantly associated with ERα expression status and persistently elevated in different stages of the breast tumor grades. High level of CASP7 expression showed better prognosis in breast cancer patients with systemic endocrine therapy as observed from Kaplan–Meier analysis. S3 and S4, estrogen responsive element (ERE) in the *CASP7* promoter, is important for estrogen-ERα-mediated CASP7 overexpression. Increased recruitment of p300, acetylated H3 and pol II in the ERE region of *CASP7* promoter is observed after hormone stimulation. Ectopic expression of *CASP7* in breast cancer cells results in cell growth and proliferation inhibition via p21^Cip^ reduction, whereas small interfering RNA (siRNA) mediated reduction of *CASP7* rescued p21^Cip^ levels. We also show that pro- and active forms of CASP7 is located in the nucleus apart from cytoplasmic region of breast cancer cells. The proliferation and growth of breast cancer cells is significantly reduced by broad-spectrum peptide inhibitors and siRNA of *CASP7*. Taken together, our findings show that CASP7 is aberrantly expressed in breast cancer and contributes to cell growth and proliferation by downregulating p21^Cip^ protein, suggesting that targeting CASP7-positive breast cancer could be one of the potential therapeutic strategies.

## Introduction

Breast cancer (BC) is a common form of cancer in women with an incidence of 232 670 new cases and 40 000 deaths estimated in 2014 in US alone (Surveillance, Epidemiology and End Results Program, National Cancer Institute). These incidence rates have declined in Caucasian women who refrained to use post menopausal hormone therapy, but have slightly risen in Afro-American cohorts. Early stages of BC development are facilitated by signals or inducers like estrogen (E2) that can orient the cells to either proliferate or differentiate.^1, 2^ Therefore, a detailed understanding and novel facets of E2 signaling may present a target for therapeutic advantage.

Majority of the BC are estrogen receptor alpha (ERα) positive, which accounts for ~60–70% of the breast cancer cases;^3, 4^ ~50% of the ER-positive BCs are estrogen dependent and responded to endocrine therapy such as tamoxifen.^5, 6, 7^ E2 acts through ERα and estrogen receptor beta (ERβ) has a pivotal role in mammary gland development and morphogenesis.^8^ E2-activated ERα regulate proliferation by regulating several cell cycle genes such as *c-Myc, cyclin D1, cyclin E, A, Cdc25A, p45Skip2* and *p27Kip1*.^9^ In addition, overexpression of ERα induces G1/S cell cycle transition by inducing *cyclin D1* and *c-Myc*^10^ and subsequent reduction of cyclin-dependent kinase inhibitors *p21*^*Cip*^ and *p27*^*Kip1*^^11^ to contribute proliferation and cell growth. Other ERs such as Orphan ERs share sequence homology and cross-talk between them induces varied cellular response.^12, 13^

Caspase 7 (*CASP7*) encodes a cysteine protease enzyme that is associated with apoptosis and inflammation.^14, 15^ CASP7 is proposed to be redundant to caspase 3 (CASP3) that has a major role in the execution of apoptosis.^16, 17^ However, recent studies have reported that CASP7 function to regulate cell cycle progression and differentiation of certain cell types.^18, 19, 20, 21^ Caspases are activated in cascades. Caspase 2, 8, 9, 10 (CASP2, 8, 9, 10) termed as initiator caspases are the large pro-domain caspases, cleave the short pro-domain caspases—caspase 3 (CASP3), 6 (CASP6) and 7 also known as effector caspases. Activated caspases have been detected in different model systems in a non-apoptotic manner.^18, 19, 22, 23^ Elevated levels of CASP7 have been reported to be associated with moderate and well-differentiated tumors in OSCC.^24^ As CASP7 was essential for proliferation and cell growth^18, 19^ and its expression in primary breast carcinoma showed no correlation with rate of apoptosis;^22^ we hypothesize that CASP7 may mediate some non-apoptotic function-like cell proliferation in the breast cancer cells. Our studies show that CASP7 is overexpressed in breast carcinoma and correlated with ERα status of the disease. We also demonstrated that overexpression of *CASP7* downregulated p21^Cip^ at the protein level and inhibition of CASP7 by broad-spectrum peptide inhibitors and small interfering RNA (siRNA) reduced the proliferation and growth of breast cancer cells.

## Results

### Aberrant *CASP7* expression in primary breast carcinoma and cell lines

**Figure 1:**
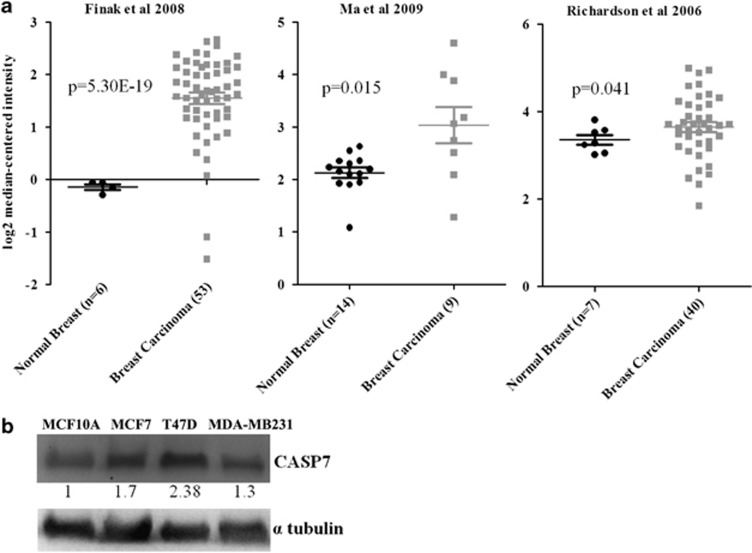
Expression of CASP7 in normal versus breast carcinoma patients and cell lines. (**a**) Increased expression of *CASP7* mRNA in breast carcinoma tissues compared with normal breast tissues in three independent Oncomine data sets (Finak *et al.*^25^; Ma *et al.*^26^; Richardson *et al.*^27^). (**b**) Elevated expression of CASP7 in breast cancer cell lines compared with normal breast cell line, MCF10A as demonstrated by western blot.

To determine the relationship between CASP7 expression and breast carcinogenesis we compared the expression of *CASP7* in normal and breast carcinoma tissues from the data obtained from Oncomine (https://www.oncomine.org). In total, 10 of the 12 data sets that contained gene expression profile of normal and breast carcinoma tissues showed elevated *CASP7* mRNA levels in primary breast carcinomas than in normal breast tissues with certain variations. The representatives of three independent data with significant *CASP7* expression were shown in Figure 1a (Finak *et al.*,^25^ *P*=5.30E-19, Ma *et al.*,^26^ *P*=0.015, Richardson *et al.,*^27^ *P*=0.041).^25, 26, 27^ Western blot result shows high CASP7 expression levels in MCF7 and T47D breast cancer cells compared with noncancerous breast cell line, MCF10A (Figure 1b), higher in MCF7 and T47D than MDA-MB231. These results show that *CASP7* is overexpressed in breast carcinoma patients and ERα-positive breast cancer cells.

### *CASP7* expression is consistently elevated in different breast tumor grades and ERα dependent

**Figure 2:**
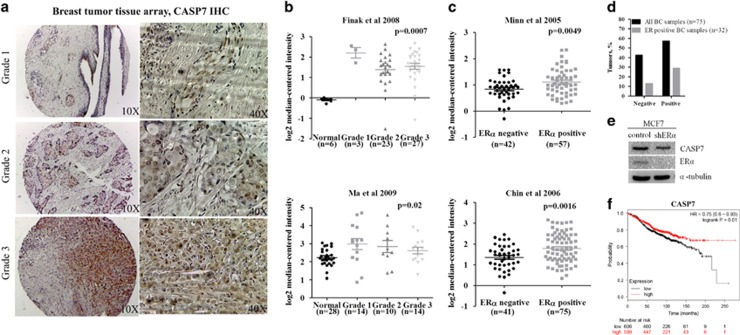
High levels of CASP7 expression in breast tumor grades and correlation with estrogen receptor status with pathological implication. (**a**) Representative immunohistochemistry images for CASP7 staining in breast carcinoma tissue microarray (TMA) with various tumor grades as specified. (**b**) Positive correlation between *CASP7* mRNA expression and tumor stages in two independent breast carcinoma data sets extracted from Oncomine (Finak *et al.*^25^; Ma *et al.*^26^). (**c**) Positive correlation between *CASP7* expression and estrogen receptor status in two independent data sets (Minn *et al.*^29^; Chin *et al.*^28^). (**d**) Percentage of CASP7 staining in breast carcinoma TMA samples. (**e**) Western blots showing efficient knockdown of ERα and reduction of CASP7 in MCF7 cells. (**f**) KM survival curve for relapse-free survival in breast cancer patients following combinatorial endocrine therapy. High expression of CASP7 correlated with better prognosis.

To assess the role of CASP7 expression in breast carcinogenesis, we analyzed commercially available tissue microarray (TMA) slide consisting of 75 breast invasive ductal carcinoma samples of different stages. Of these 75 samples, 32 (42.66%) were negative and 43 (57.34%) were positive for CASP7 staining (Table 1). Analysis by grades showed no correlation with CASP7 expression; however, a consistent high level of CASP7 expression was observed in different stages of the breast carcinoma (Figure 2a, Table 1). Similarly, the *CASP7* expression was analyzed in Oncomine data sets where the breast carcinomas were grouped according to different stages of the disease. Finak *et al.*^25^ and Ma *et al.*^26^ data sets indicated a significant upregulation of *CASP7* mRNA in different grades of breast carcinoma (Figure 2b). In addition, a significant correlation between *CASP7* mRNA expression and ERα positivity in breast carcinoma tissues of Chin *et al.*^28^ and Minn *et al.*^29^ data sets was observed (Figure 2c). TMA analyses showed 29% positive and no variation (21% negative, 20% positive) in CASP7 expression in ERα-positive and -negative breast tumors, respectively (Table 1, Figure 2d). The results were further confirmed in ERα knockdown MCF7 cells, which showed a significant decrease in CASP7 expression with ERα depletion in the cells (Figure 2e). To further examine the clinicopathological implication of CASP7 expression in the breast cancer patients, a cancer survival analysis was performed using Kaplan–Meier plot (http://www.kmplot.com/breast/). High expression of CASP7 showed better relapse-free survival (HR=0.75 (0.6–0.93), *P*=0.01) in ER-positive breast cancer patients with combinatorial endocrine therapy (Figure 2f). No prognostic value of CASP7 expression was seen in tamoxifen-treated (Supplementary Figure 1a) and ER-negative (Supplementary Figure 1b) breast cancer patients. These results illustrate for the first time that CASP7 expression has a significant clinical relevance and provide probability to predict relapse-free survival in breast cancer.

### *CASP7* overexpression is estrogen dependent

**Figure 3:**
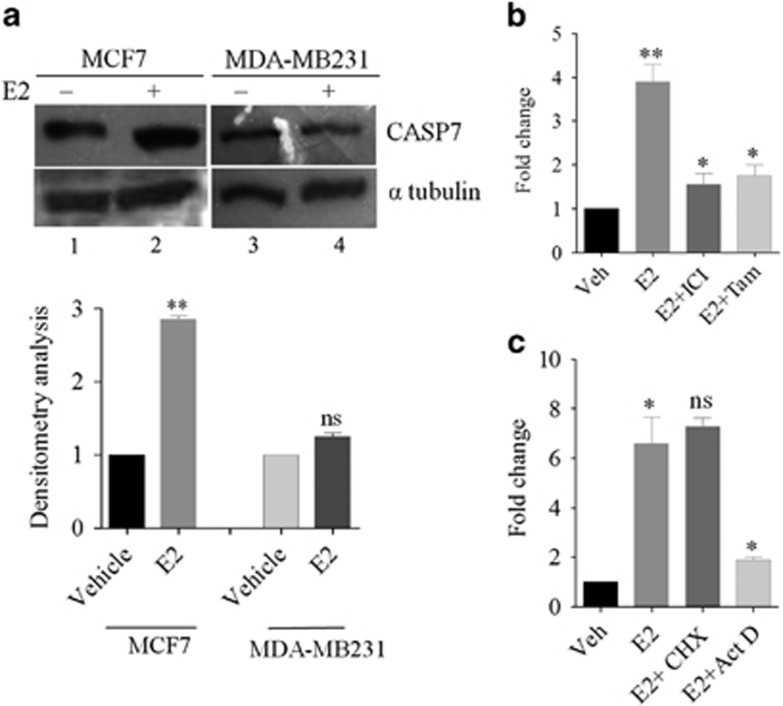
Estrogen regulates CASP7 expression. (**a**) Western blot showing CASP7 expression in MCF7 and MDA-MB231 cell line under E2 influence (upper panel) and densitometry (lower panel). (**b**) Quantitative RT–PCR showing *CASP7* mRNA expression after treatment with E2 and E2 inhibitors- ICI182,780 and tamoxifen. (**c**) Quantitative RT–PCR showing *CASP7* mRNA expression with protein synthesis and transcription inhibitors, cyclohexamide and actinomycin D, respectively. **P<0.01. *P<0.05. ns=no significant change.

As CASP7 expression was found to correlate with ERα, we sought to investigate the role of E2, a ligand of ERα on CASP7 expression. To examine this, ER-positive cell line, MCF7 and ER-negative cell line, MDA-MB231 were treated with 100 nM of E2. E2 treatment after 48 h, western blot analysis showed a significant upregulation of *CASP7* in MCF7 cells (~1.9-fold) compared with control cells but had no effect in MDA-MB231 cells (~1.3-fold) (Figure 3a). Tamoxifen (10 μM) and ICI 182 780 (1 μM) treatment in MCF7 cells significantly inhibited the E2-induced *CASP7* mRNA levels (Figure 3b). To understand whether E2 affects directly, protein synthesis inhibitor, cyclohexamide (CHX, 10 μg/ml) was added prior to hormone treatment. The *CASP7* induction by E2 was irresponsive to this treatment, suggesting the effect to be protein synthesis independent. However, treatment with transcription inhibitor, actinomycin D (5 μM) significantly inhibited E2-induced expression (Figure 3c). These results show that *CASP7* expression is E2 stimulated and is not mediated by induced proteins.

### Presence of estrogen responsive elements in the *CASP7* promoter

**Figure 4:**
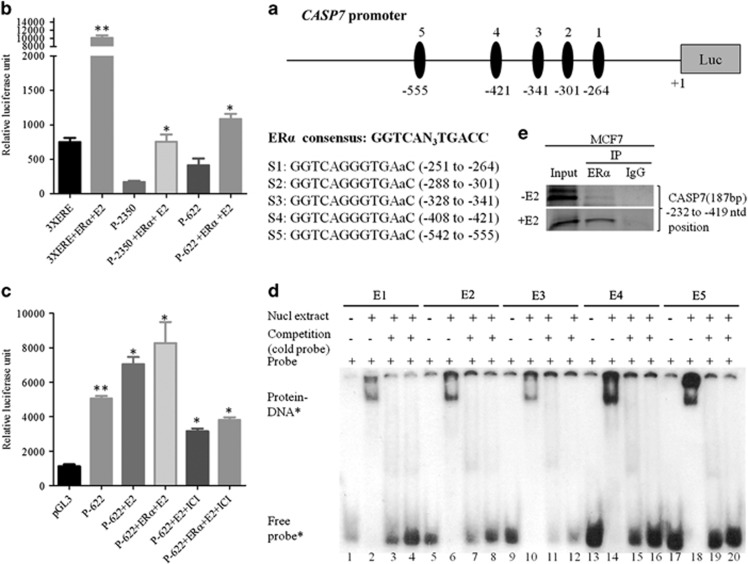
ERα classically regulates *CASP7* promoter. (**a**) Schematic diagram of *CASP7* promoter (upper panel) and putative ERα-binding sites (lower panel). (**b**) Relative luciferase activities after co-transfecting *CASP7* promoters (P-2350 & P-622) and *ERα* in MCF7 cells after 48 h. The data shown are the means±s.e. of three individual experiments. (**c**) Relative luciferase activities of P-622 construct with ERα co-transfection after hormone and ICI treatments. The data shown are the means±s.e. of three individual experiments. (**d**) *In vitro* DNA–protein-binding assay using 30 base pair oligonucleotides containing putative ERE sites of *CASP7* promoter. Cold competition reaction included 50–100-fold molar excess of consensus ERE sequence. ‘−' and ‘+' signifies with and without nuclear lysate, respectively. (**e**) *In vivo* DNA–protein-binding assay of ERα in the putative ERE sites present in the *CASP7* promoter in the presence or absence of E2. **P<0.01 and *P<0.05.

Next, to understand the E2-mediated upregulation of CASP7 we analyzed the *CASP7* promoter. *In silico* analysis of *CASP7* promoter demonstrated the presence of five putative estrogen responsive elements (ERE) (S1: −251/−264, S2: −288/−301, S3: −328/−341, S4: −408/−421, S5: −542/−555) upstream of the transcription start site (Figure 4a). Previously established *CASP7* promoter,^30^ a kind gift from Professor Srikumar P Chellappan (H Lee Moffitt Cancer Center and Research Institute, FL, USA) and a 622 base pair (–622/+1) *CASP7* promoter subcloned into pGL3 luciferase vector termed as P-2350 and P-622, respectively, were subjected to dual luciferase assay after 48 h of co-transfecting the promoter constructs with *ERα* (gift from Professor Ratna K Vadlamudi, University of Texas Health Science Center, San Antonio, TX, USA) in MCF7 cells. E2 significantly upregulated the luciferase activities of P-2350 and P-622 by approximately four and ~2.6-fold, respectively, compared with untreated cells (Figure 4b). However, the luciferase activity of P-622 was ~1.5-fold higher than P-2350 construct during *ERα* co-transfection and hormone stimulation (Figure 4b). Therefore, P-622 construct was used for further luciferase experiments. Moreover, the luciferase activity of P-622 was approximately ninefold lower than *3XERE* control luciferase construct containing three consensus ERE sites. ICI182,780 significantly reduced the E2-induced *CASP7* luciferase activity (Figure 4c).

To test the binding of ERα to the putative ERE sequences, we performed electrophoretic mobility shift assay with oligonucleotides containing putative EREs using nuclear extract from MCF7 cells. The electrophoretic mobility shift assay oligonucleotides containing ERE elements were named as E1 (−244/−276 for S1), E2 (−281/−311 for S2), E3 (−325/−354 for S3), E4 (−399/−429 for S4) and E5 (−534/−564 for S5). The nuclear protein formed complexes with all the radiolabelled double-stranded oligonucleotides (Figure 4d). The complexes were completely abolished with 50- or 100-fold molar excess of unlabeled double-stranded ERE consensus sequences. *In vivo* chromatin immunoprecipitation assays (ChIP) assay was performed using antibody specific to ERα in MCF7 cells in the presence or absence of E2. Significant levels of ERα were recruited to the ERE sites during E2 treatment compared with untreated samples (Figure 4e). The PCR product (−232/−419) from immunoprecipitated DNA covered four response elements (S1, S2, S3, S4). Site-specific ChIP PCRs were not performed owing to the close proximity of the response elements and a presence of 40 base pair repeat sequence (−281/−328 and −328/−368) adjacent to each other in the *CASP7* promoter. The exact function of this repeat sequence is not known. These data indicate that *CASP7* promoter is regulated ‘classically' by ERα.

### Other ERs also regulate *CASP7* promoter

**Figure 5:**
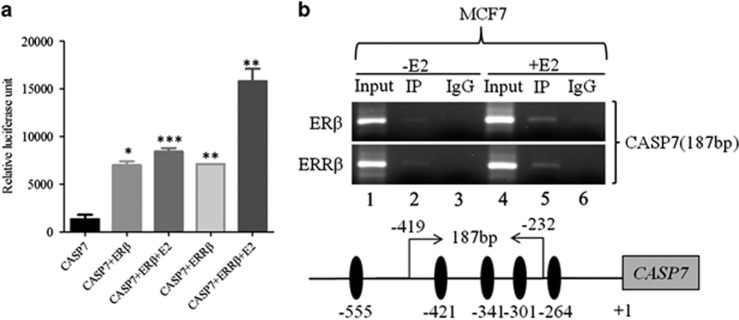
Other estrogen receptors can also regulate *CASP7*. (**a**) Relative luciferase activities of *CASP7* after co-transfecting P-622, *ERβ* and *ERRβ* with subsequent E2 treatment for 24 h. (**b**) *In vivo* binding of ERβ and ERRβ in the *CASP7* promoter through putative ERE sites with and without E2 treatment (upper panel). Schematic diagram of Chip PCR performed in *CASP7* promoter (lower panel). *P<0.05. **P<0.01. ***P<0.001.

As ERs, ERβ and ERRβ, share sequence homology and recognize ERE to control overlapping pathways,^12, 31, 32^ we tested the effect of these receptors on *CASP7* promoter. The P-622 promoter construct was co-transfected with *ERβ* and *ERRβ* (gift from Professor Ratna K Vadlamudi and Professor Philippa Saunders, MRC Human Reproductive Sciences Unit, UK) with subsequent E2 treatment for 24 h. Dual luciferase assay after 24 h showed a significant P-622 promoter activity with *ERβ* and *ERRβ* co-transfection. Interestingly, *ERRβ* induced luciferase activity of *CASP7* was ~1.9-fold greater than *ERβ* owing to E2 stimulation (Figure 5a). We next evaluated the physical interaction of ERβ and ERRβ with the *CASP7* promoter using ChIP assay. Significantly, the E2 treated MCF7 cells showed increased recruitment of ERβ and ERRβ compared with untreated control cells (Figure 5b). Taken together, we observed that ERβ and ERRβ also regulate *CASP7* promoter.

### Site 3 and 4 is critical in ERα-mediated *CASP7* promoter regulation

**Figure 6:**
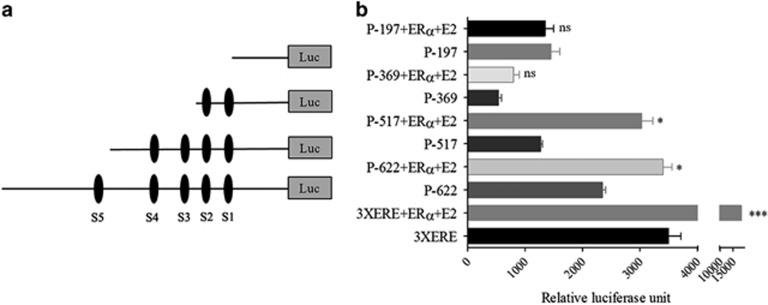
Site 3 and 4 are major response element for E2-mediated *CASP7* transcription. (**a**) Schematic diagram of *CASP7* deletion constructs. (**b**) The relative luciferase activity of different deletion constructs of *CASP7* after post co-transfecting with *ERα* and E2 treatment for 48 h. The data shown are the means±s.e. of three individual experiments. *P<0.05. ***P<0.001. ns=no significant change.

Next, to identify which of the five ERE elements in the *CASP7* promoter was important, different deletion constructs- P-517 (−517/+1, S5 deletion), P-369 (−369/+1, S5 and S4 deletion) and P-197 (−197/+1, all ERE deletion) were co-transfected with *ERα* in MCF7 cells followed by E2 treatment for 24 h (Figure 6a). S2 and S3 deletion was not possible owing to the presence of 40 base pair repeat sequences adjacent to them. The luciferase activities of P-622 and P-517 were significantly upregulated (P-622, *P*<0.05 and P-517, *P*<0.05) as a result of E2 treatment compared with control cells (Figure 6a). Compared with P-517 construct (S5 deletion) the luciferase activity of P-369 construct (S4 and S3 deletion) was significantly reduced (*P*<0.01). Although a slight nonsignificant elevation of luciferase activity of P-369 was detected with co-transfection and treatment. Taken together, luciferase assays with the deletion constructs suggest S3 and S4 to be important for ERα-mediated *CASP7* transactivation.

### p300 upregulates *CASP7* promoter activity via acetylating histone H3

**Figure 7:**
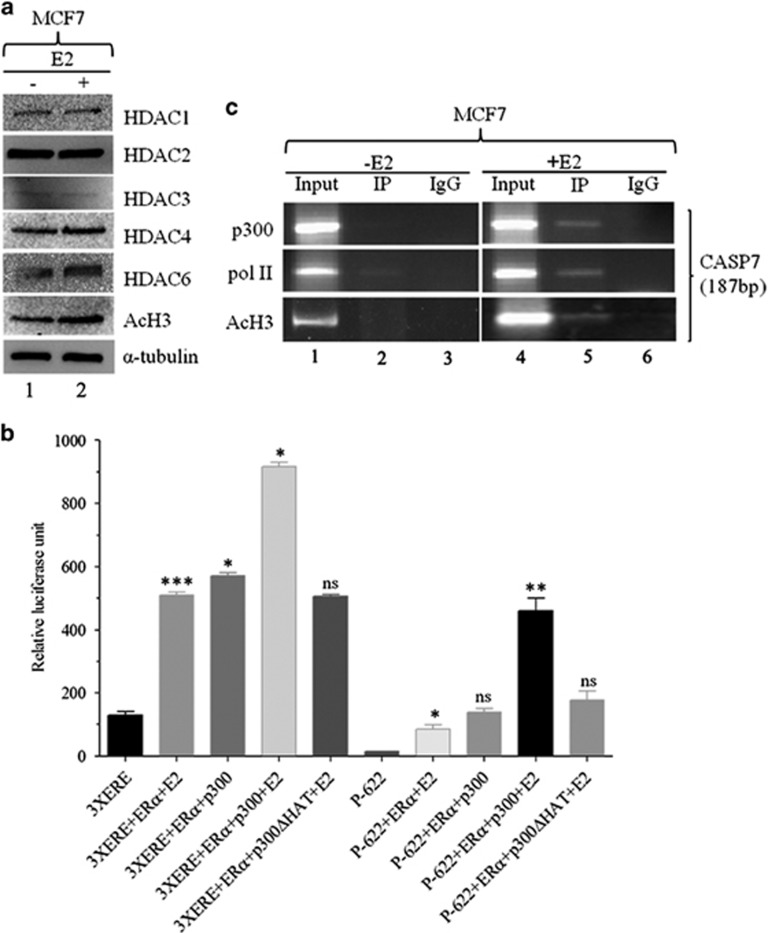
Epigenetic factors positively regulate *CASP7* transcription. (**a**) Western blot of different HDACs and acetylated H3 from whole-cell lysate extracted from MCF7 cells treated with E2 for 48 h. (**b**) Relative luciferase activities of *CASP7* promoter post co-transfection with *p300*, *p300ΔHAT* and *ERα* after 24 h of E2 treatment. (**c**) Recruitment of p300, pol II and acetylated H3 quantified by Chip PCR in the presence (right panel) or absence (left panel) of E2. *P<0.05. **P<0.01. ***P<0.001.

Changes in gene expression and protein activities have an important role in cancer initiation and progression, part of which is controlled by histone deacetylases (HDACs) that affect cell growth, differentiation and apoptosis.^33^ Therefore, we examined if E2 stimulation had any effect on HDAC expression. We found that E2 stimulation in MCF7 cells did not have any significant effect on the HDAC protein levels (Figure 7a). However, the acetylated histone 3 levels were significantly upregulated during treatment compared with control cells. The acetylation of H3 involves transcriptional coactivator, p300,^34^ which is a component of ERα coactivator complex.^35^ Subsequently, *p300* (gift from Dr Joan Boyes, Institute of Cancer Research, London, UK) and *ERα* plasmids were co-transfected with P-622 luciferase construct in MCF7 cells and treated with E2 for 24 h. The *3XERE Luc* was used as a positive control. Significantly, co-transfection of *ERα* and *p300* drastically increased the luciferase activity of P-622. In contrast, significant reduction in P-622 and *3XERE Luc* luciferase activities were observed when wild-type *p300* was substituted with histone acetyltransferase domain deleted *p300* plasmid (*p300ΔHAT*, gift from Dr Joan Boyes), indicating that acetylation of H3 is important for *CASP7* regulation (Figure 7b). We further determined whether p300 and other factors were enriched in the *CASP7* promoter during treatment by ChIP experiments. ChIP assay showed that p300, pol II and acetylated histone 3 proteins were enriched in the *CASP7* promoter during treatment compared to control cells (Figure 7c). These results show CASP7 expression is controlled by an ERα coactivator component, p300 by changing acetylated H3 levels.

### Overexpression of *CASP7* may regulate cell growth and proliferation via downregulating p21^Cip^

**Figure 8:**
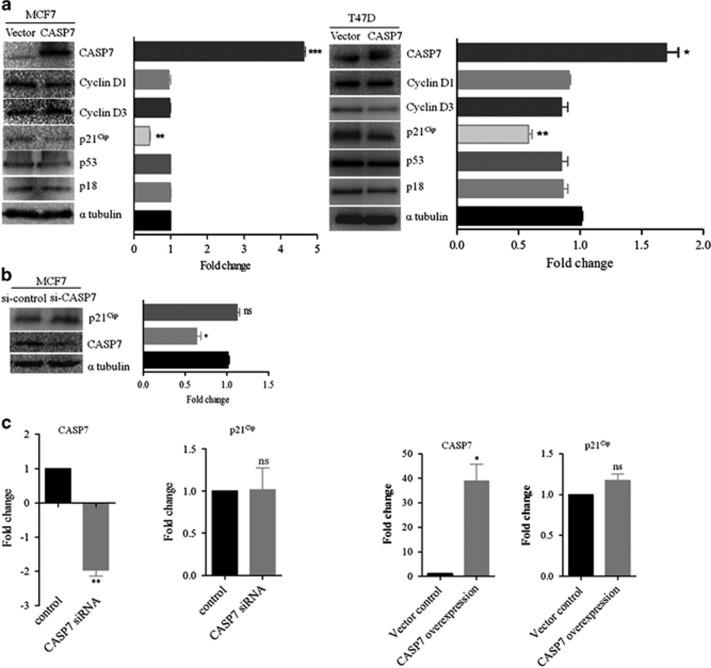
CASP7 targets p21^Cip^. (**a**) Western blot of different cell cycle proteins performed after transfecting *CASP7* expression vector in MCF7 and T47D cell lines (left panel). Right panel depicts densitometric analyses of the western blot performed in MCF7 and T47D. (**b**) Western blots performed in whole-cell extract isolated from *CASP7* siRNA-transfected MCF7 cells (left panel). Right panel illustrates densitometric analyses of the western blot data. (**c**) MCF7 cells were transfected with *CASP7* siRNA (100 pico mole) and vector expressing *CASP7*. Forty-eight hours later, the expression levels of *CASP7* and *p21*^*Cip*^ were evaluated by using quantitative RT–PCR. *P<0.05. **P<0.01. ***P<0.001.

Earlier reports have demonstrated the non-apoptotic role of CASP7 targeting various cell cycle inhibitors and DNA repair proteins.^36, 37^ Our previous experiments showed the overexpression of CASP7 in breast carcinomas; therefore, to determine whether CASP7 affects the cell cycle, we overexpressed *CASP7* (gift from Dr Jean-Bernard Denault, Université de Sherbrooke, Sherbrooke, QC, Canada) in MCF7 and T47D cells and examined the cell cycle proteins by western blot. *CASP7* overexpression was found to significantly reduce the expression of cell cycle inhibitor, p21^Cip^ in both the breast cancer cells (MCF7 ~2.5-fold; T47D ~1.6-fold) (Figure 8a). However, no significant changes were observed in other cell cycle proteins examined. In addition, transient siRNA mediated knockdown of *CASP7* mRNA abrogated CASP7 mediated p21^Cip^ reduction (Figure 8b), suggesting its effect on cell growth and proliferation. CASP7 has been demonstrated to target p21^Cip^ protein at caspase cleavage motif *DHVDL* yielding fragments with a molecular weight ~12.4 and 6 KDa.^38^ Therefore, it becomes apparent to investigate whether the p21^Cip^ reduction was due to protein cleavage or transcript level decrease. To address this issue, we isolated total protein and RNA from the *CASP7* overexpressed MCF7 cells and only RNA from siRNA *CASP7* knockdown samples. Interestingly, overexpression and knockdown of CASP7 did not change the *p21*^*Cip*^ mRNA levels (Figure 8c), suggesting that the reduction may be possible only at protein levels (Figure 8a). However, though several attempts were made to detect the cleaved forms of p21^Cip^ in the stripped blots overexpressed with CASP7 but it was undetected. As CASP7 expression is E2 dependent and cleaved forms of CASP7 was detected only in cancerous cell lines and not in MCF10A (Supplementary Figure 4) we next asked a question if E2 treatment or ectopic expression of *ERα* leads to activation of CASP7 to affect p21^Cip^. Western blot performed after 48 h of *ERα* transfection and E2 treatment clearly indicates a decreased level of p21^Cip^ protein (Supplementary Figure 2, lane 2, 3, 5, 6). However, we could not detect the cleaved forms of p21^Cip^ although protein level reduction was observed. No significant change in the activated forms of CASP7 was seen despite E2 treatment or ERα transfection (Supplementary Figure 2) suggestive of activation pathway not dependent on hormone stimulation. Taken together, our results show that CASP7 downregulates p21^Cip^ at the protein level.

### CASP7 and its active forms are localized in the nucleus apart from cytoplasmic region

**Figure 9:**
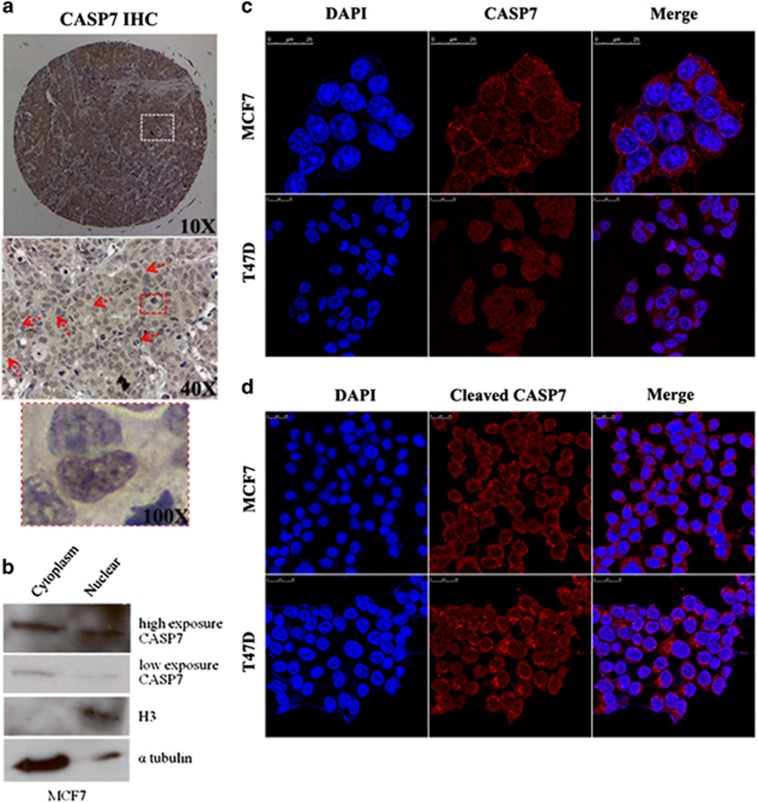
Localization of CASP7 and cleaved CASP7 in the nucleus. (**a**) TMA of breast carcinoma is stained with CASP7 antibody (ab32522). Arrow mark shows positive staining of CASP7 in the nucleus. (**b**) Western blot showing localization of CASP7 in the nucleus performed after cell fractionation of MCF7 cells. H3 and α tubulin are used to confirm the fractionation. (**c**, **d**) Localization of CASP7 and cleaved CASP7 was determined by indirect immunofluorescence using primary antibodies against CASP7 and cleaved CASP7 and alexa flour-conjugated antigoat and antirabbit secondary antibodies in MCF7 and T47D cell lines. DNA was stained with DAPI.

As CASP7 downregulates p21^Cip^ to inhibit proliferation and growth of the cells,^39^ it was worth to detect the localization of CASP7 in the cells. Earlier reports suggested CASP7 localization in both cytoplasm and nucleus;^21, 40, 41^ however, not much is known in the breast cancer cells. To investigate the localization of CASP7, we analyzed the TMA data of breast carcinoma, which show presence of CASP7 in the nucleus apart from cytoplasmic region of the cells to possibly regulate nuclear proteins (Figure 9a). We also performed cell fractionation and antibody staining of CASP7 protein in breast cancer cells. As shown in Figure 9b, cell fractionation analysis confirmed the presence of CASP7 in the nucleus with ~30% of the cellular CASP7 based on western blot data. Confocal analysis showed a punctate-like structure of CASP7 and its active form in both nucleus and the cytoplasm of MCF7 and T47D cells (Figure 9c). The specificity of immunofluorescent staining and presence of CASP7 and its cleaved forms were confirmed by the lysates fractionated from MCF7 and T47D cells. The cytoplasmic fraction of both the cell lines show a small amounts of active form of CASP7 but not in nucleus, which might be due to low amounts in the nucleus (Supplementary Figure 3). It was also essential to know if the activated forms of CASP7 exist in the nontransformed breast epithelial cell line, MCF10 or only in breast cancer cells. Western blot from the whole-cell lysate from MCF10A and MCF7 clearly suggest the presence of activated forms of CASP7 in the latter but not in MCF10A (Supplementary Figure 4).

### Broad-spectrum caspase inhibitors and small interfering mediated *CASP7* knockdown affect proliferation and cell growth

**Figure 10:**
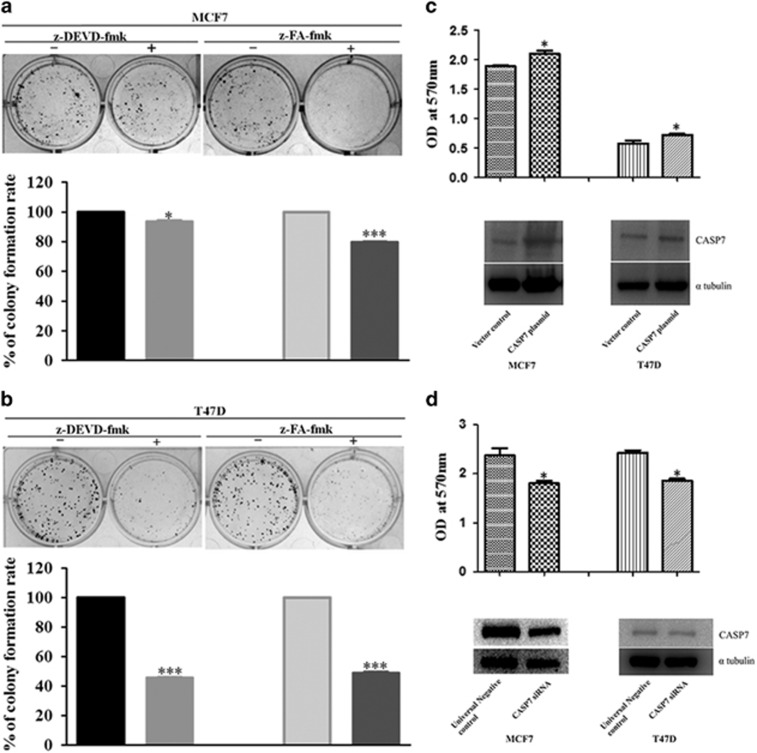
Effect of broad-spectrum caspase inhibitors and CASP7 on proliferation and growth of breast cancer cells. (**a**, **b**) Colony-forming ability of MCF7 and T47D cells after treatment with caspase inhibitors: z-DEVD-fmk (10 μmol/l) and z-FA-fmk (30 μmol/l). Growth of cells (>50 cells/colony) was stained by measured by 0.1% (w/v) crystal violet solution and measured by dissolving the dye with 10% (v/v) acetic acid at 590nm. (**c**, **d**) Proliferation of breast cancer cells were tested by transient transfection of *CASP7* expression vector (300 ng) and siRNA (100 pmole) in MCF7 and T47D. Transfection efficiency was quantified by western blot with CASP7 antibody (1:5000, abcam) and α tubulin (1:10000, sigma) was used as an internal control. Proliferation was checked by MTT assay. The data shown (means±s.e.) were obtained from three individual experiments. *P<0.05. ***P<0.001.

We next investigated the effect of peptide inhibitors of CASP7 on proliferation and growth of breast cancer cells. The sensitivity of breast cancer cells to peptide inhibitors, z-DEVD-fmk and z-FA-fmk was checked by MTT assay at a concentration below 100 μmol/l. A significant decrease in the growth of breast cancer cells (MCF7 and T47D) was observed at a concentration of 10 μmol/l and 30 μmol/l in case of z-DEVD-fmk and z-FA-fmk-treated cells, respectively, from colony-forming assay (Figures 10a and b). As the peptide inhibitors used also have effect on other caspases apart from CASP7, which are also proved to have a role in proliferation, we performed MTT assay after transient overexpression or knockdown of *CASP7*. Transient overexpression or knockdown of *CASP7* after 48 h showed a significant change in the proliferation of breast cancer cells (Figures 10c and d, upper panels). The increased proliferation of breast cancer cells was reversed upon knockdown of *CASP7* (Figure 10d). The overexpression and knockdown of *CASP7* was confirmed by western blot (Figures 10c and d, lower panels). Taken together, results from peptide inhibitors and knockdown experiments suggest the probably role of CASP7 in cell proliferation and growth of breast cancer cells.

## Discussion

In this paper, we demonstrated that CASP7 overexpression in primary breast carcinoma is ERα dependent and its high expression is correlated with better prognosis. We also provide evidence that CASP7 and its active form are localized in the nucleus apart from cytoplasm and regulate p21^Cip^ expression. Furthermore, inhibition of CASP7 by broad-spectrum peptides significantly reduced the proliferation and growth of breast cancer cells. Therefore, our study proposes that CASP7 may function as one of the factors to contribute breast carcinogenesis.

Elevated levels of CASP7 are seen to associate with moderate and well-differentiated tumors in Oral Squamous Cell Carcinoma.^24^ Although knockdown or inhibition of CASP7 leads to decreased proliferation and growth of different cancerous cells.^18, 19^ Expression studies of CASP7 in breast carcinoma are not much known. A single literature study in primary breast carcinoma showed that expression of CASP7 had no correlation with the rate of apoptosis, suggesting its role other then apoptosis.^22^ Our studies from Oncomine data sets showed a significant upregulation of *CASP7* mRNA expression in BC patients compared with the normal breast tissues, which was further confirmed in breast cancer cell lines. The upregulation of *CASP7* expression showed a positive correlation with ERα presence as revealed from Minn *et al.* and Chin *et al.* data sets, which was also confirmed in ERα knockdown cells (Figures 2c and e). Such aberrant expression of CASP7 was also seen in different grades of the breast carcinoma as observed from TMA and Oncomine data sets though with certain variations. Such variations in CASP7 expression were also detected in other cancers,^42^ which might be due to the genetic and epigenetic factors associated with the disease. Our initial experiments and analyses showed that CASP7 might be involved in breast carcinogenesis. However, the molecular mechanism of the aberrant CASP7 expression in BC is yet unknown. Kaplan–Meier survival curves for relapse-free survival indicated a better prognosis with higher expression of CASP7 in patients with combinatorial endocrine therapy and no survival significance in patients treated exclusively with tamoxifen or in ER-negative BC patients. These findings indicate the potential need for combinatorial therapy in BC patients with high CASP7 expression.

Majority of the breast cancers are ERα dependent (~70%) and E2 driven.^43, 44^ Genome-wide analysis of ERα recruitment to the target promoter post E2 treatment demonstrated a wide array of genes regulated directly or indirectly by ERα.^45, 46^ Notably, both the genome-wide analysis identified *CASP7* as a direct target gene of ERα but its molecular regulation was not well defined. This study provides a novel mechanism underlying E2-mediated CASP7 induction and also its implications in breast cancer cells. All the five putative cis-acting ERE elements in *CASP7* promoter had the ability to recruit ERα but only ERE sites, S3 (−328/−341) and S4 (−408/−421) have the potential to regulate E2-mediated ERα transactivation of *CASP7* promoter. Distinct effects of E2 on the interaction of ERs with other proteins may lead to differential activity of ERs. ERs belong to a large superfamily of nuclear receptors and share a significant sequence homology with the orphan nuclear receptor.^12^ Therefore, cross-talk between the receptors, ERβ and ERRβ, by binding to the full-length or extended half ERE sites recognized by ERα cannot be ignored.^12, 31, 32^ In support to this mechanism, increase in ERβ and ERRβ levels positively regulate the *CASP7* promoter during E2 treatment (Figure 5a). ERRβ is known to constitutively activate transcription without any ligand.^47^ However, ectopic expression of *ERRβ* in the presence of E2 significantly increased the transcriptional activity of *CASP7* probably due to the physical interaction between ERα and ERRβ during treatment.^48^ Consequently, the physical occupancy of ERα as a homodimer and with ERRβ as a heterodimer in the response element of *CASP7* during hormone stimulation is one the major factor for the enhanced *CASP7* transcription.

Gene expression is maintained by a critical balance of histone acetylation and deacetylation pattern induced by histone acetyltransferases and HDACs, respectively. Aberrant expression of HDACs is associated with tumor development^49, 50, 51, 52, 53, 54^ by inducing altered gene expression linked to proliferation, cell cycle and apoptosis. No significant change in the HDACs levels with E2 treatment was observed. However, certain variation in HDAC expression was observed in breast cancer TMA, which correlated with hormone receptor status.^55^ Moreover, significant upregulation of acetylated H3 and its recruitment along with ERα and p300 in the presence of hormone resulted in pol II occupancy in the *CASP7* promoter to increase its transcription. The result was consistent with previous studies in the dorsal hippocampus.^34, 35, 56^

Further investigating the role of CASP7 overexpression on cell cycle proteins in breast cancer cells revealed that CASP7 significantly reduced p21^Cip^ levels and not other proteins. This phenomenon was reversed by transient knockdown of *CASP7*. Earlier reports revealed that CASP7 target p21^Cip^ by cleaving at *DHVDL* motif (amino-acid positions—109 and 114) consequently yielding fragments with molecular weight 12.4 and 6 kDa.^38^ Hence, we speculated that p21^Cip^ downregulation during *CASP7* overexpression was a result of proteolytic cleavage of the protein and not transcript reduction. Though p21^Cip^ protein level was downregulated with *CASP7* overexpression or E2 treatment we are still unsure if it was due to proteolytic cleavage as cleaved forms were not detected after repeated experiments. Although detectable levels of activated CASP7 were observed in breast cancer cells (Figure 9d, Supplementary Figure 2) with little change during hormone stimulation or ERα overexpression might suggest the mechanism of induction and activation of CASP7 to be an independent phenomena. To our surprise, activated forms of CASP7 in MCF7 cells was detected but not in noncancerous breast epithelial cells, MCF10A (Supplementary Figure 3). Therefore, it will be intriguing to understand whether the active forms of CASP7 are specific to only cancerous cells as observed in our studies. The presence of cleaved forms of CASP7 along with other active caspases has been demonstrated in cervical cancer cell line, HeLa.^18^ The upstream activated caspases like CASP8 may be responsible for activating CASP7 in breast cancer cells (data not shown) or HeLa cell line.^18^ Though p21^Cip^ levels were decreased after E2 treatment or ERα ectopic expression but it is not clear if the downregulation was mediated through CASP7 as E2 is also known to downregulate p21^Cip^ expression.^11, 57^ CASP7 seen to localize in the cytoplasmic and nuclear compartments executes apoptosis by cleaving several endogenous substrates such as p21^Cip^, p27^Kip1^, MCM3 and so on.^58, 59^ In accordance with previous studies,^22^ we also observed positive CASP7 staining in both nucleus and cytoplasm in primary breast carcinoma though certain variations were observed. The localization of active form of CASP7 was speculated previously in the nucleus to regulate cell cycle progression, which the authors did not confirm its presence.^19^ In our present study we could detect the active form of CASP7 by immunofluorescence in the nucleus of MCF7 and T47D cell lines but its presence was hardly detected in the nucleus during fractionation assay of breast cancer cells, which might be probably due to levels lower in the nucleus than in cytoplasm. However, the localization of active form of CASP7 in the nucleus cannot be ignored as it was observed during immunofluorescence. Active forms of CASP7 have the potential to target p21^Cip^ thereby negatively regulate cell cycle, inhibit cell growth and proliferation through binding to PCNA or inhibition of CDK2 activity.^60, 61^

Overexpression and activation of CASP7 in BC cell lines underlined the possible activation processes facilitated by initiator caspases, CASP8 and CASP9.^62^ The cleavage of poly-(ADP-ribose) polymerase 1, hallmark of CASP3/CASP7 activation^63^ was not detected despite the presence of activated CASP8 and CASP7 in the BC cells (data not shown). Inhibition of CASP7 by caspase inhibitors- z-DEVD-fmk and z-FA-fmk could significantly decrease the proliferation and growth potential of the breast cancer cells. As the CASP7 inhibitors used were broad-spectrum inhibitors; therefore, offset effect of other caspases cannot be ignored. However, MCF7 cells are CASP3 deficent^64^ and the presence of activated CASP7 in an apoptosis independent manner to downregulate p21^Cip^ suggests its role in proliferation, but our results do not deny the role of other possible caspases in proliferation of breast cancer cells. Altogether, our results advocate the possible role of CASP7 in breast cancer progression by targeting p21^Cip^ that may be considered as an alternative therapeutic target against breast cancer.

## Materials and methods

### Tissue microarray

BC tissue microarray slide (Cat No. BR1505) was purchased from US Biomax (Rockville, MD, USA) and stained with CASP7 antibody (ab32522, Abcam, Cambridge, MA, USA) as previously described.^65^ The images were captured by Leica (Wetzlar, Germany). The slide was examined and scored by an experienced pathologist. The scoring was performed based on intensity of positive staining and percentage of cells positive for the stain.

Intensity score: 0 −ive, 1+ mild, 2+ moderate, 3+ strong.

Percentage of tumor cell positive: 0–100%.

### Cell culture and treatment

MCF7, T47D and MDA-MB231 cell lines purchased from NCCS (Pune, India) were maintained as previously described.^66^ MCF10A, a kind gift from Dr Annapoorni Rangarajan (IISc, Bangalore, India) was maintained as previously described.^67^ The cells maintained for 48–72 h in phenol-free medium prior to hormone treatments were then treated with estrogen (Sigma, St Louis, MO, USA) at a concentration of 100 nM for 24–48 h. During inhibition studies, MCF7 cells were treated with 10 μM of tamoxifen and 1 μM of ICI182,780 (Sigma) for 6 h prior to hormone treatment. To study the direct effect of estradiol in CASP7 expression, MCF7 cells were treated with 10 μg/ml of cyclohexamide and 5 μM of actinomycin D (Sigma) for 30 min followed by hormone treatment for 24–48 h.

### RNA isolation and quantitative reverse transcriptase PCR

Total RNA was isolated from the cells using Trizol reagent (Sigma). A total of 200 ng of DNase I-digested RNA was used to generate complementary DNA using first strand DNA synthesis kit (Life Technologies, Carlsbad, CA, USA). Quantitative PCR was performed using CASP7-specific primers and glyceraldehyde-3-phosphate dehydrogenase was used as internal control (Supplementary Table 1). ΔΔCt values were calculated and plotted from three independent experiments.

### Plasmids, cell transfection and luciferase assay

For stable knockdown cell line generation, *ERα* (Sigma) was transfected in MCF7 cells using ICAFectin 441 (Eurogentec, Seraing, Belgium). After 24 h post transfection, the cells were subjected to puromycin selection (2 μg/ml) (Life Technologies). Transient knockdown of *CASP7* was performed by transfecting 100 pmoles of siRNA clusters (*CASP7*-antisense sequences: 5′-UUUGCUGAAUCCUCAACCC-3′, 5′-AUGUUGUACUGAUAUGUAG-3′, 5′-UUCUUCUCAUGGAGGUGUG-3′) (Eurogentec) in MCF7 cells. Universal negative control siRNA (Eurogentec) was used as a mock. siRNAs were transfected by using ICAFectin 442 (Eurogentec). For luciferase assays, MCF7 cells seeded in 24-well plates were grown upto 60% confluency and then transfected with 100 ng of luciferase constructs, 200 ng of the expression vector and 10 ng of the renilla luciferase internal control using ICAFectin 441. Twenty-four hour post transfection, the cells were treated with 100 nM of estrogen or 1 μM ICI182,780 for additional 20 h. Cells were then lysed and Dual Luciferase assay (Promega, Madison, WI, USA) was performed as per manufacturer's instructions. *3XERE luc*, a gift from Dr Doris Germain (Mount Sinai Cancer Institute, New York, USA) was used as positive control for estrogen-stimulation assays. Statistical analyses were carried out by using Graph Pad Prism 5.01 software. Unpaired *t-*test was carried out to compare between the two groups. *P-*values<0.05 were considered statistically significant. The primers used for generating deletion constructs are provided in the Supplementary Table 1.

### Electrophoretic mobility shift assay

Electrophoretic mobility shift assay was performed as described previously.^68^ Oligonucleotides with putative ERα-binding sites were synthesized as provided in the Supplementary Table 1. Competition assays included 50–100-fold molar excess of ERα cold consensus oligonucleotide.

### Chromatin immunoprecipitation assay

ChIP assays were performed as described previously.^69^ The antibodies used for ChIP assay includes normal IgG mouse (C15400001) or rabbit (C15410206, Diagenode, Seraing, Belgium), ERα (8644) and acetylated histone 3 (9649, CST, Danvers, MA, USA), ERβ (2780-1) and ERRβ (ab19331, Abcam), p300 (sc-584) and Pol II (sc-899, Santa Cruz, Dallas, TX, USA). The immunoprecipitated DNA samples were PCR amplified using primers mentioned in Supplementary Table 1.

### Western blot

Western blots were performed using whole-cell lysates extracted using radioimmunoprecipitation assay buffer and as described previously.^68^ Nuclear and cytoplasmic extracts were fractionated using a CelLytic NuCLEAR Extraction Kit (Sigma) following manufacturer's instructions. Antibodies used include α-tubulin (T5168, Sigma), ERα (8438), cyclin D1 (2978), cyclin D3 (2936), p21^Cip^ (2947), HDAC1 (5356), HDAC2 (5113), HDAC3 (3949), HDAC4 (7628), HDAC6 (7558), acetylated histone 3 (9649), p18 (2896) and cleaved CASP7 (8438, CST), CASP7 (ab32522, Abcam), H3 (sc-8654), p300 (sc-584) and pol II (sc-899, Santa Cruz) and p53 (550832, BD Biosciences, East Rutherford, NJ, USA). Cleaved CASP7 (8438) detects both cleaved and uncleaved CASP7. Images were acquired by using Chemidoc XRS+ molecular 228 imager (Bio-Rad, Hercules, CA, USA). Western blot images were quantified using Image J software (NIH, Bethesda, MD, USA).

### Cell proliferation assay

Cells (5 × 10^3^ cells/well) grown either in Dulbecco's Modified Eagle Medium or Roswell Park Memorial Institute medium 1640 with 10% fetal bovine serum at 37 °C for 24–48 h in 24-well plates. Approximately 300 ng of CASP7 expression vector or vector control was transfected using ICAFectin 441 (Eurogentec). For CASP7 knockdown 100 pmole of siRNA was transfected and Universal negative control used as a mock was transfected using ICAFectin 442 (Eurogentec). Both expression vector and siRNA-transfected breast cancer cells were incubated for another 48 h. After 48 h of incubation, 3-(4 5-dimethylthiazol-2-yl)-2 5-diphenyltetrazolium bromide assay was performed as described previously^70^ and measured at 570 and 620 nm by using Varioskan Flash Multimode Reader (Thermo Scientific, Waltham, MA, USA). Each experiment was performed atleast three times in triplicates.

### Colony-forming assay

For anchorage-dependent colony-formation assay, ~2.5 × 10^2^ cell/well were seeded in a 12-well plate (BD Biosciences). MCF7 and T47D cell lines were treated with CASP7 inhibitors at a concentration below 100 μmol/l.^71, 72^ z-DEVD-fmk and z-FA-fmk at a concentration of 10 μmol/l and 30 μmol/l, respectively, was added to breast cancer cells and maintained at 37 °C in a humidified condition of 5% CO_2_. Fresh complete growth medium was added with indicated inhibitor concentrations after every 72 h for 8 days. The cells were then washed with PBS (phosphate-buffered saline), fixed with 10% formalin for 10 min and stained with 0.1% (w/v) crystal violet solution for 20 min at room temperature. Excess dye was removed by extensive washing with deionized water and air dried prior to solubilization of bound dye in 100 μl of 10% (v/v) acetic acid. The solubilized dye was measured at 590 nm using Varioskan Flash Multimode Reader (Thermo Scientific).

### Immunofluorescence

Approximately 0.3 × 10^6^ cells were seeded in 0.2% (w/v) gelatin (Sigma)-coated glass coverslips. At 60–70% confluency, the cells were washed thrice with 1 × PBS, fixed with methanol/acetone (1:1) and incubated for 30 min to 1 h at −20 °C. Cells were blocked with blocking buffer (1 × PBS, 0.1% (w/v) bovine serum albumin, 0.3% (v/v) Triton X-100), incubated with corresponding primary antibodies CASP7 (ab32522, Abcam) and cleaved CASP7 (sc-22179, Santa Cruz) overnight and stained with subsequent Alexa fluor secondary antibodies (Life Technologies) for 40 min. The cleaved CASP7 antibody (sc-22179) detects only the cleaved form of CASP7 protein. Cells washed with Tris-Buffered Saline and Tween 20 and stained with DAPI (Life Technologies) were mounted on glass slides with prolong antifade reagent (Life Technologies). Images were obtained by confocal microscope (Leica).

## Figures and Tables

**Table 1:** CASP7 staining in TMA

	*CASP7 staining*	
	*Negative*	*Positive*
Patients	32 (42.66%)	43 (57.34%)

*Receptor subtype*		
ER+/PR+	8 (10.66%)	17 (22.66%)
ER−/PR+	1 (1.33%)	0
ER+/PR−	2 (2.66%)	5 (6.66%)
ER-/PR−	20 (26.66%)	20 (26.66%)

*Histological grade*		
Grade I	2 (2.66%)	5 (6.66%)
Grade II	25 (33.33%)	31 (41.33%)
Grade III	6 (8%)	6 (8%)

Abbreviations: CASP7, caspase 7; ER, estrogen receptor; PR, progesterone receptor; TMA, tissue microarray.
