# Supplementary material for: Overexpression of caspase 7 is ERα dependent to affect proliferation and cell growth in breast cancer cells by targeting p21Cip
Source: Oncogenesis. 2016 Apr 18;5(4):e219–. doi: 10.1038/oncsis.2016.12 (PMC4848833; doi:10.1038/oncsis.2016.12)
Supplement: Supplementary Table 1 [file oncsis201612x1.doc]

Supplementary Figure 1. KM survival curves for relapse free survival with tamoxifen endocrine and combinatorial therapy in ER positive (a) and ER negative (b) breast cancer patients respectively.

Supplementary Figure 2. Effect of E2 and ERα on CASP7 and p21^Cip^. The western blot was performed in whole cell lysates (15-20 μg) extracted after 48 h of E2 treatment or ERα transient transfection. Lane 2 and 5 shows the overexpression of ERα ( indicate ERα fused with GFP). Cleaved CASP7 antibody (8438, CST) was used to detect both the active and pro- forms of CASP7 protein (* indicate cleaved form). The blots were stripped with stripping buffer (Thermo Scientific) and reprobed with subsequent antibodies. α tubulin was used as an internal control. The dilution of the antibodies used are- ERα (1:1000), p21^Cip^ (1:1000), Cleaved CASP7 (1:1000), α tubulin (1:10000). The densitometry of p21^Cip^ bands are quantified by ImageJ software.

Supplementary Figure 3. Localization of cleaved CASP7 in breast cancer cells. Western blot of cleaved CASP7 was performed in cytoplasmic and nuclei fractionated lysates of MCF7 and T47D cell lines. Histone 3 (H3) and α tubulin was used a control to determine contamination.

Supplementary Figure 4. Expression of cleaved CASP7 in noncancerous breast epithelial cells, MCF10 and cancerous MCF7 cells. Western blots of cleaved CASP7 and ERα was performed in whole cell lysates (15-20 μg) extracted from MCF10A and MCF7 cell lines using RIPA buffer. * indicates cleaved form of CASP7 protein. The antibody dilution used are- ERα (1:1000), cleaved CASP7 (1:1000, CST) and α tubulin (1:10000). α tubulin was used as an internal control to check equal loading.
