# Supplementary figures and images for: Overexpression of caspase 7 is ERα dependent to affect proliferation and cell growth in breast cancer cells by targeting p21Cip
Source: Oncogenesis. 2016 Apr 18;5(4):e219–. doi: 10.1038/oncsis.2016.12 (PMC4848833; doi:10.1038/oncsis.2016.12)

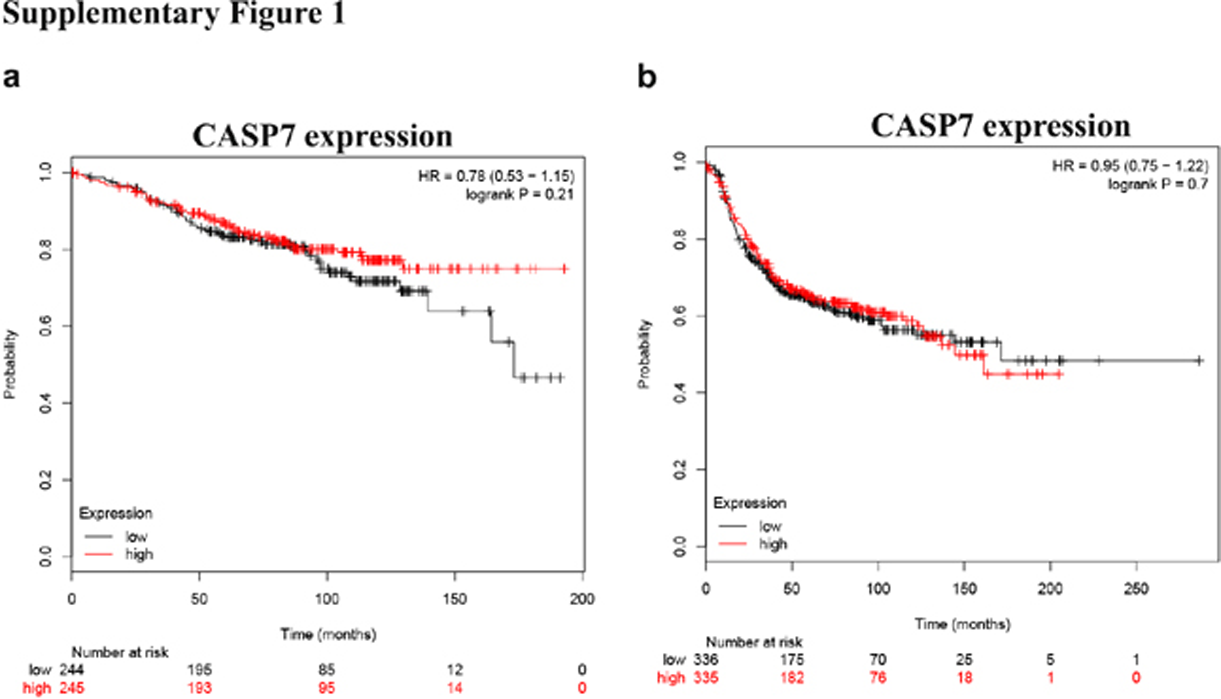

Supplement: Supplementary Figure 1 [file oncsis201612x2.tif]

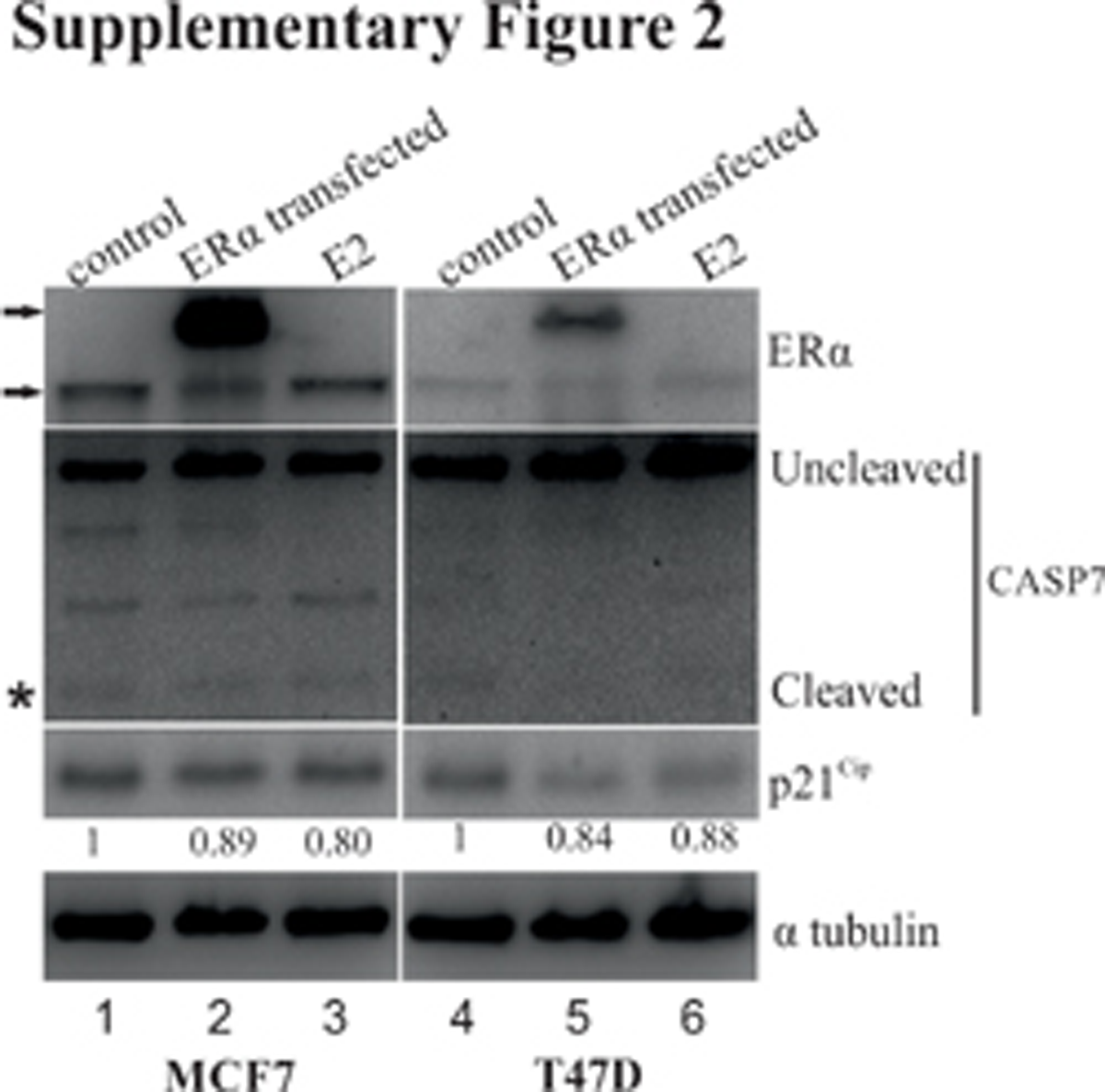

Supplement: Supplementary Figure 2 [file oncsis201612x3.tif]

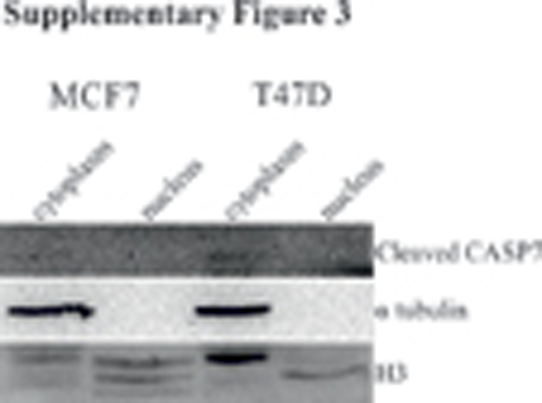

Supplement: Supplementary Figure 3 [file oncsis201612x4.tif]

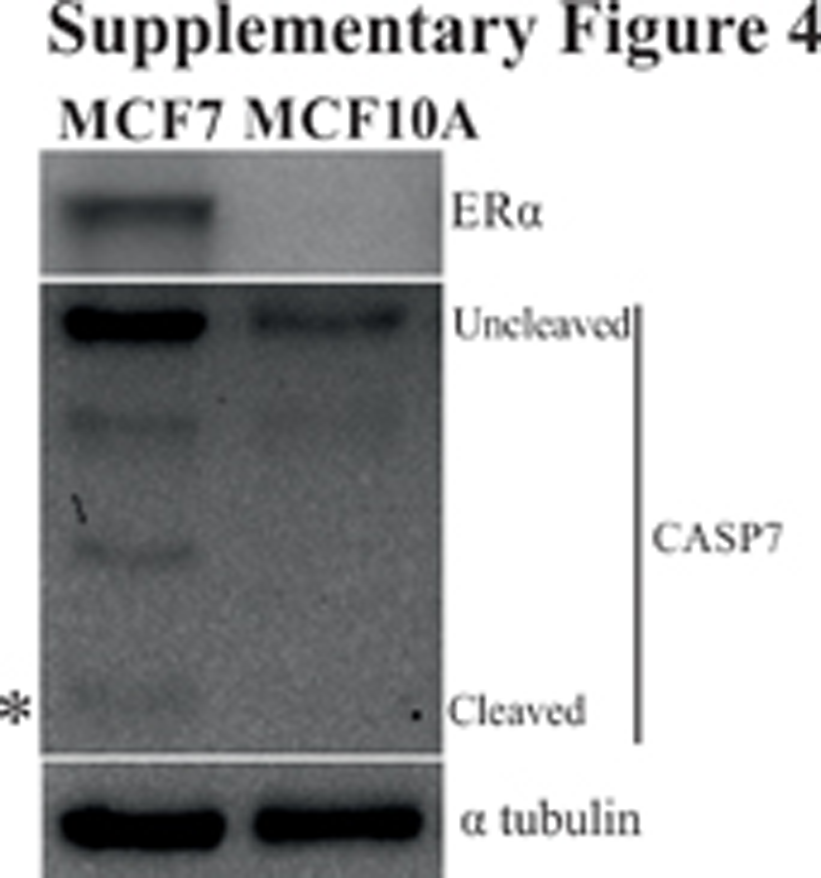

Supplement: Supplementary Figure 4 [file oncsis201612x5.tif]
